# *Cdh1* functions as an oncogene by inducing self-renewal of lung cancer stem-like cells via oncogenic pathways

**DOI:** 10.7150/ijbs.38672

**Published:** 2020-01-01

**Authors:** Ting Ye, Jingyuan Li, Zhiwei Sun, Doudou Liu, Bin Zeng, Qiting Zhao, Jianyu Wang, H. Rosie Xing

**Affiliations:** 1 Institute of Life Sciences, Chongqing Medical University, Chongqing, China; 2 Laboratory of Translational Cancer Stem Cell Research, Chongqing Medical University, Chongqing, China; 3 College of Biomedical Engineering, State Key Laboratory of Ultrasound Engineering in Medicine, Chongqing Medical University, Chongqing, China; 4 Department of Laboratory Medicine, the Affiliated Hospital of Southwest Medical University, Luzhou, China

**Keywords:** cancer stem cells, *Cdh1*, oncogene, self-renewal, oncogenic pathways

## Abstract

The mortality rate of lung cancer remains the highest amongst all cancers despite of new therapeutic developments. While cancer stem cells (CSCs) may play a pivotal role in cancer, mechanisms underlying CSCs self-renewal and their relevance to cancer progression have not been clearly elucidated due to the lack of reliable and stable CSC cellular models. In the present study, we unveiled the novel oncogene function of cadherin 1 (*Cdh1*) via bioinformatic analysis in a broad spectrum of human cancers including lung adenocarcinoma (LUAD), adding a new dimension to the widely reported tumor suppressor function of *Cdh1*. Experimentally, we show for the first time that *Cdh1* promotes the self-renewal of lung CSCs, consistent with its function in embryonic and normal stem cells. Using the LLC-Symmetric Division (LLC-SD) model, we have revealed an intricate cross-talk between the oncogenic pathway and stem cell pathway in which *Cdh1* functions as an oncogene by promoting lung CSC renewal via the activation of the Phosphoinositide 3-kinase (PI3K) and inhibition of Mitogen-activated protein kinase (MAPK) pathways, respectively. In summary, this study has provided evidence demonstrating effective utilization of the normal stem cell renewal mechanisms by CSCs to promote oncogenesis and progression.

## Introduction

Lung cancer is currently the most common cause of cancer related death worldwide 1. Among all primary lung cancers, non-small cell lung cancer (NSCLC) accounts for 85% of all lung carcinomas and lung adenocarcinoma (LUAD) is the main pathological subtype 2, 3. Despite of therapeutic developments, the prognosis and treatment efficacy of LUAD have not been substantially improved, generally attributed to late diagnosis and tumor metastasis. As such, the 5-year survival rate remains less than 15% 4, 5. Thus, search for new molecular mechanisms associated with LUAD oncogenesis and progression is ongoing. Accumulating evidence suggest lung cancer stem-like cells (CSCs), a group of cells with self-renewal capability, may play a role in lung cancer initiation and progression 6, 7. However, the cancer biology characteristics of CSCs have not been well elucidated due to the lack of stable cellular models of CSCs. Consequently, mechanisms that regulate CSC self-renewal are poorly defined 8, 9.

*Cdh1* gene coding E-cadherin is a transmembrane calcium-dependent adhesion molecule expressed in almost all epithelial cells 10. In addition, E-cadherin is highly conserved evolutionarily, and is crucial for embryonic stem cell pluripotency, self-renewal and differentiation 11-16. In early cancer literature, *Cdh1* is widely regarded as a tumor suppressor gene 17. It's down-regulation or silencing by DNA methylation is associated with loss of epithelial morphology and increased invasiveness through epithelial-mesenchymal transition (EMT) 18-20 and is correlated with high grade, advanced stage, and poor prognosis 21, 22. Noteworthy, in recent years, few studies showed a positive correlation between *Cdh1* expression and metastasis 23-25, though the mechanisms explored appeared to involve the reserve process of EMT - MET (mesenchymal to epithelial transition) 26-28. The discrepancies between these findings and those on *Cdh1* as a tumor suppressor have not been resolved. In addition, whether *Cdh1* may regulate the self-renewal of CSCs as it does in normal stem cells has not been examined at the mechanistic level.

Bioinformatics has been widely applied in cancer research. In the present study, through bioinformatics analyses of Oncomine, Gene Expression Omnibus (GEO), The Cancer Genome Atlas (TCGA) and Gene Expression Profiling Interactive Analysis (GEPIA) databases, we uncovered that *Cdh1* gene expression was elevated in human cancer tissues compared with normal counterparts in 17 types of cancers analyzed, including LUAD. Moreover, in LUAD, *Cdh1* expression correlated with clinicopathological features and prognosis. This clinical finding has added a new dimension to our knowledge about *Cdh1* in addition to its role as a tumor suppressor. Moreover, *Cdh1* expression was increased in the mouse LLC-SD lung adenocarcinoma CSC cellular model we generated. Using the LLC-SD model, we have revealed an intricate cross-talk between the oncogenic pathway and stem cell pathway in which *Cdh1* functions as an oncogene by promoting lung CSC renewal via the activation of the PI3K and inhibition of MAPK pathways, respectively. Further, we show for the first time that *Cdh1* promotes the self-renewal of lung CSCs, consistent with its function in embryonic and normal stem cells. In summary, this study has provided new evidence demonstrating the effective utilization of the normal stem cell renewal mechanisms by CSCs to promote oncogenesis and progression.

## Materials and Methods

### Bioinformatics analysis of *Cdh1*

The mRNA expression of *Cdh1* in a variety of tumor types was analyzed by GEPIA database (http://gepia.cancer-pku.cn/index.html). The Oncomine datasets (https://www.oncomine.org/) were used to analyze the expression of *Cdh1* in LUAD tumors. Students' t-test was used, and two-times of fold change with the P-value of <0.0001 was defined as clinically significant. All of the data from the TCGA-LUAD datasets (https://cancergenome.nih. gov/) were downloaded including the *Cdh1* mRNA expression levels and clinicopathological features of tumor staging. Human lung adenocarcinoma data was extracted from the GEO database, accession numbers GSE32867 29 (n = 57 patients) dataset. Meanwhile, the following datasets were included as Bild dataset 30 and Selamat dataset 29 from the Oncomine database. The association between the expression of *Cdh1* and survival, including overall survival (OS), progression-free survival (PFS) and post-progression survival (PPS) was assessed through analysis in the Kaplan-Meier plotter (http://kmplot. com/analysis/).

### Cell culture and cell lines

LLC-Parental cell line was purchased from the Cell Bank of the Chinese Academy of Sciences (Shanghai, China) and cultured in Dulbecco modified Eagle medium (DMEM) high glucose medium (Hyclone, USA) containing 10 % FBS (Gibco, USA). LLC-SD cells, the stem-cell component of the LLC-Parental 31, were maintained in serum-free DMEM-F12 medium (Hyclone, USA) containing B27 Supplement (Gibco, USA).

### Reverse transcription and quantitative real-time polymerase chain reaction (RT-qPCR)

For RT-qPCR experiments, total RNA was isolated using TRIZOL (Takara, Japan) and reverse-transcribed into cDNA following to the manufacturer's instructions. Relative expression was normalized to that of TBP internal control. The following PCR condition was used on the Light Cycler: 39 cycles of 95°C for 30s, 95°C for 5s, followed by 60°C for 30s in a 10µl reaction volume. The primer sequences for RT-qPCR are listed in Table 3.

### Western blotting (WB)

Cells were lysed in RIPA (Radio Immunoprecipitation Assay) buffer (Beyotime, China) containing 1% PMSF. Extracted protein concentration was measured by BCA (bicinchoninic acid) method and stored at -80°C. 25 µg of protein sample was resolved by 8-10% SDS-PAGE and transferred to a PVDF membrane (Bio-Rad, USA). Afterwards, the membrane was blocked in 5% nonfat milk (BD, USA) and incubated with primary antibodies at 4°C overnight. Finally, the membrane was incubated with secondary antibodies and developed under the gel electrophoresis imager (Bio-Rad, USA). GAPDH protein was used as a reference control. The following primary antibodies were used: anti-E-cadherin (Proteintech, USA), anti-PI3K, anti-Akt, anti-mTOR, anti-S6K, anti-Eif4e, anti-Erk1/2, anti-p-Mek1/2, anti-p-Erk1/2, anti-p-Akt, anti-p-mTOR, anti-p-S6k (Cell Signaling Technology, USA), anti-Mek1 (Upstate, USA) and anti-GAPDH (Proteintech, USA).

### siRNA transient interference assay

The siRNAs were synthesized and purchased from GenePharma Company (Shanghai, China). 1×10^6^ LLC-SD cells were seeded in each well of a six-well culture plate (Thermo, USA). 5µl of siRNA combined with 5µl of Lipofectamine 2000 (Thermo, USA) were diluted in 200µl MEM medium (Gibco, USA), respectively, and incubated for 5 min. The mixture was then used for transient interference assay following the manufacturer's protocol. The sequences of siRNAs are as follows: 5'-UUCUCCGAACGUGUCACGUTT-3' (siN.C.), 5'-AGUCGGACAUCUGGAGCAUTT-3'(siMek1/2), 5'-GGAGAGACAUCUACGAAAUTT-3'(sip110), 5'-GGGUCAGGAAAUCACAUCUTT-3'(siCdh1).

### Soft agar colony formation assay

Cells were seeded into the medium containing 0.2% agar (Sigma, USA) in 6-well cell culture plates at a density of 200 cells/well and cultured for 1 weeks. The colonies that consisted of more than 50 cells were then stained with 0.05% crystal violet, counted and recorded.

### Single cell colony formation assay

Single cell suspension was prepared and the concentration was adjusted to 10 cells/ml. One hundred microliters of cell suspensions were seeded into each well of 96-well plates (Thermo, USA). Single-cell seeding was confirmed using microscope and wells containing one single cell were marked. After cultured at 37°C and 5% CO_2_ for 10 days, colonies exceeding 50 cells were counted.

### Protein-protein interaction (PPI) network analysis

The 50 most frequently altered neighbor genes analyzed from cBioportal of *Cdh1*. STRING database version 11.0 (http://www.string-db.org) was used to analyze PPI network of the neighbor genes of *Cdh1*. PPI refers to the protein complex formed by two or more proteins through covalent bond. The most important protein and connected nodes were analyzed by R-3.51 software.

### Gene Ontology (GO) enrichment analysis

To investigate the intrinsic mechanisms of *Cdh1* expression in LUAD tissues, we acquired *Cdh1*-associated co-expression genes from cBioPortal database, the GEPIA database and the UALCAN database (http://ualcan.path.uab.edu/index.html). This study selected genes with an absolute value of the Pearsons' correlation coefficient >0.4 in LUAD. Finally, the sum aggregate results among cBioPortal, GEPIA and UALCAN were obtained and used for further analyses. GO enrichment analysis and functional annotation for co-expression genes were performed using KOBAS 3.0 (https:// kobas.cbi.pku.edu.cn/), which is used to evaluate the significance of enrichment of pathways in human tumors.

### Gene set enrichment analysis (GSEA)

GSEA was performed using GSEA 3.0 software. 594 LUAD samples in TCGA were divided into a low expression group and high expression group using *Cdh1* expression median level as a cut-off point. In order to identify the potential functions of *Cdh1*, GSEA was used to determine whether a series of previously-defined gene sets were enriched in different phenotypes. The expression level of *Cdh1* (high versus low) was used as the phenotypic label and the number of permutations was 1000. The gene expression datasets were used collections H (Hallmark gene set) and C2 (curated gene set: KEGG), publicly available at MsigDB (http://www.broad.mit.edu/gsea/msigdb/index.jsp). All other parameters were set to default values. A nominal P-value of *P* < 0.05 and false discovery rate (FDR) < 0.25 were chosen as significance cut-off criterion.

### Serial spheroid formation assay

For spheroid formation, 1000 cells were mixed with 2ml culture medium, and plated into each well of 6-well plate. Cells were photographed and counted after 5 days of growth and diluted to a density of 500 cells/ml for reseeding. The assay was repeated for three rounds.

### Construction and transfection of lentiviral vectors expressing shRNA

The shRNA that targets the sequence of the optimal siRNA was designed and synthesized. The annealed oligonucleotide fragment was cloned into the lentivirus plasmid PLL3.7(Addgene, USA) to establish the shRNA lentiviral vector. The recombinant plasmid DNA was identified by sequencing. When the cultured 293T cells reached a 70-80% density, Lipofectamine 2000 was used to mediate transfection of shRNA lentiviral plasmid DNA with the right sequence, medium was renewed at 12h. The incubation was continued for 48h, when the supernatant of each culture was collected and filtered by 0.22 µm filter (Millipore, USA). Following concentration, the supernatant was stored in aliquots at -80°C. Lentivirus infections of LLC-SD cell lines were carried out with the presence of 8 µg/ml of polybrene.

### Animals

BALB/c nude mice (6-8 weeks old) were obtained from Huafukang bioscience company (Beijing, China). C57BL/6 mice (6-8 weeks old) were obtained from Chongqing national biological industry base experimental animal center of Chongqing Medical University. All animal studies were conducted in accordance with the approved protocol by the Institutional Review of Chongqing Medical University and carried out in accordance with the animal welfare and institutional ethical guidelines of Chongqing Medical University.

### Subcutaneous tumor transplantation assay in BALB/c nude mice

For transplantation analysis uaing BALB/c nude mice, approximately 1 × 10^3^ single cell suspensions were mixed with 50μL Matrigel Matrix (Corning) at a 1:1 ratio and 100μL mixture was subcutaneously injected into the both insides of the hind legs of mice. Tumor size was assessed every two days and tumor volume was calculated using V= (length x width^2^)/2. The mice were euthanized and killed when the tumor volume reached about 1000mm^3^.

### Orthotopic tumor transplantation in C57BL/6 mice

Single cell suspensions (1×10^5^) mixed with 12.5μL Matrigel Matrix (Corning) at a 1:1 ratio was injected subcutaneously into left lobe of the lungs of C57BL/6 mice as we previously described 31. For tumorigenesis analysis, the mice were sacrificed, orthotopic and metastatic lung nodules were counted and immunohistochemistry was evaluated after 14 days. For the survival assay, the death time of each mouse was monitored and recorded after tumor transplantation.

### Statistical analysis

Data was analyzed by Student's independent t-test of variance using GraphPad Prism and R-3.51 software and described as mean ± SEM indicated in figure legends. Differences were considered statistically significant when *P* < 0.05. *P < 0.05, **P < 0.01, *** P < 0.001.

## Results

### The expression of *Cdh1* is elevated in human cancer tissues including lung adenocarcinoma

**Figure 1:**
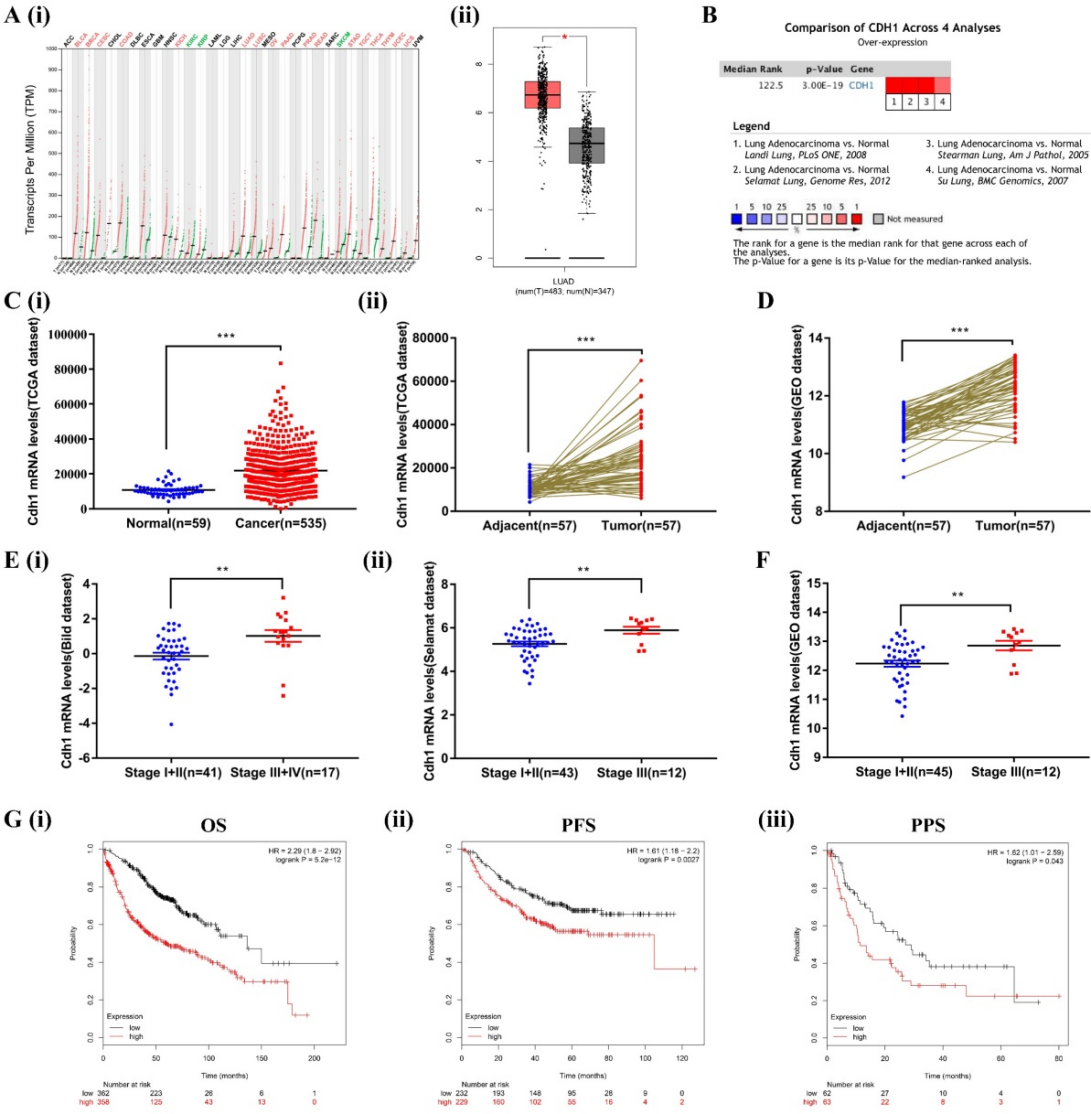
** The bioinformatic data mining analysis of prognostic significance of *Cdh1* expression. (A)(i)** *Cdh1* gene expression profile across all types of tumor samples and normal tissues from GEPIA analysis. **(ii)** The differential expression of *Cdh1* mRNA between LUAD tissues (n=483) and corresponding control samples (n=347) from GEPIA analysis. Red box is LUAD tissues (T), the gray box is the normal tissues (N). **(B)** The combined results of *Cdh1* expression across four studies in ONCOMINE datasets, the redder the square, the more is the correlation with cancer state. **(C)(i-ii)** *Cdh1* expression level in LUAD cancer tissues is higher than that in the normal or pericarcinomatous tissues. The mRNA levels of *Cdh1* in unmatched LUAD and matched LUAD were download from TCGA datasets. **(D)** The mRNA levels of *Cdh1* in LUAD and matched non-cancerous lung tissue based on GEO LUAD dataset GSE32867. **E(i-ii)** Correlation between *Cdh1* expression and tumor stage in LUAD patients by analysis of the Oncomine datasets (Bild and Selamat dataset). **(F)** Differential expression of *Cdh1* in early and late tumor stage of LUAD patients based on GSE32867.** (G) (i-iii)** Elevated expression of *Cdh1* indicated poor clinical outcome for LUAD patients. The overall survival (OS), progression-free survival (PFS) and post-progression survival curves of LUAD patients with high (red) or low (black) *Cdh1* expression were plotted from Kaplan-Meier plotter database. (* *p* < 0.05, ** *p* < 0.01, *** *p* < 0.001).

We first compared the transcriptional levels of *Cdh1* in 33 types of cancers with their normal counterparts by using GEPIA database (http://gepia.cancer- pku.cn/). *Cdh1* expression was increased in 17 types of cancers, decreased in 3 types of cancers, and there was no significant difference in 13 types of cancers (**Figure 1A-i**). These findings, for the first time revealed that elevated *Cdh1* expression is a rather common feature of human cancer that is currently under appreciated. We conduced in-depth analysis in LUAD. For LUAD, *Cdh1* transcriptional level in tumor tissues (483 cases) was significantly elevated compared with the normal lung tissues (347 cases) (**Figure 1A-ii**). To validate the results of the GEPIA database, we first conducted *Cdh1* expression analyses using the Oncomine (www.oncomine.org) database for the expression of *Cdh1* in lung adenocarcinoma. Four datasets were included in the analysis: the 'Landi Lung' 32, 'Selamat Lung' 29, 'Stearman Lung' 33 and 'Su Lung' 34. *Cdh1* was significantly overexpressed in LUAD compared to normal individuals (**Figure 1B**). Similar findings in LUAD were obtained from additional analyses performed using the TCGA database (LUAD group: n = 535 cases; normal group: n = 59 cases) (**Figure 1C-i**), and the TCGA and GEO database (LUAD tissues: n = 57 cases; adjacent normal tissues: n = 57 cases) (**Figure 1C-ii and Figure 1D**). These results indicated that *Cdh1* expression is elevated in human cancer, including lung adenocarcinoma.

### Increased expression of *Cdh1* is associated with TNM stage and poor prognosis in human lung adenocarcinoma

To verify the relationship between the level of expression of *Cdh1* and clinicopathological features of patients with LUAD, we further analyzed the expression of *Cdh1* with TNM using Oncomine (the Bild dataset^30^ and the Selamat dataset^29^) and the GEO database. Interestingly, *Cdh1* expression level was much higher in patients with advanced stage (stageⅢ~Ⅳ) disease compared with those with early stage (stageⅠ~Ⅱ) disease (**Figure 1E and Figure 1F**). Kaplan-Meier Plotter tool (http://kmplot.com/ analysis/) was used to analyze the correlation between the *Cdh1* expression and LUAD survival. LUAD patients with a low *Cdh1* expression exhibited a better overall survival (OS) [HR = 2.29, P < 0.001] (**Figure 1G-i**), progression-free survival (PFS) [HR = 1.61, P = 0.0027] (**Figure 1G-ii**) and post-progression survival (PPS) [HR = 1.62, P = 0.043] (**Figure 1G-iii**) compared with patients with a high *Cdh1* expression. Thus, a low *Cdh1* expression appears to be a protective factor and a favorable prognostic marker for patients with LUAD.

### *Cdh1* regulates self-renewal of LLC-SD cells *in vitro*

**Figure 2:**
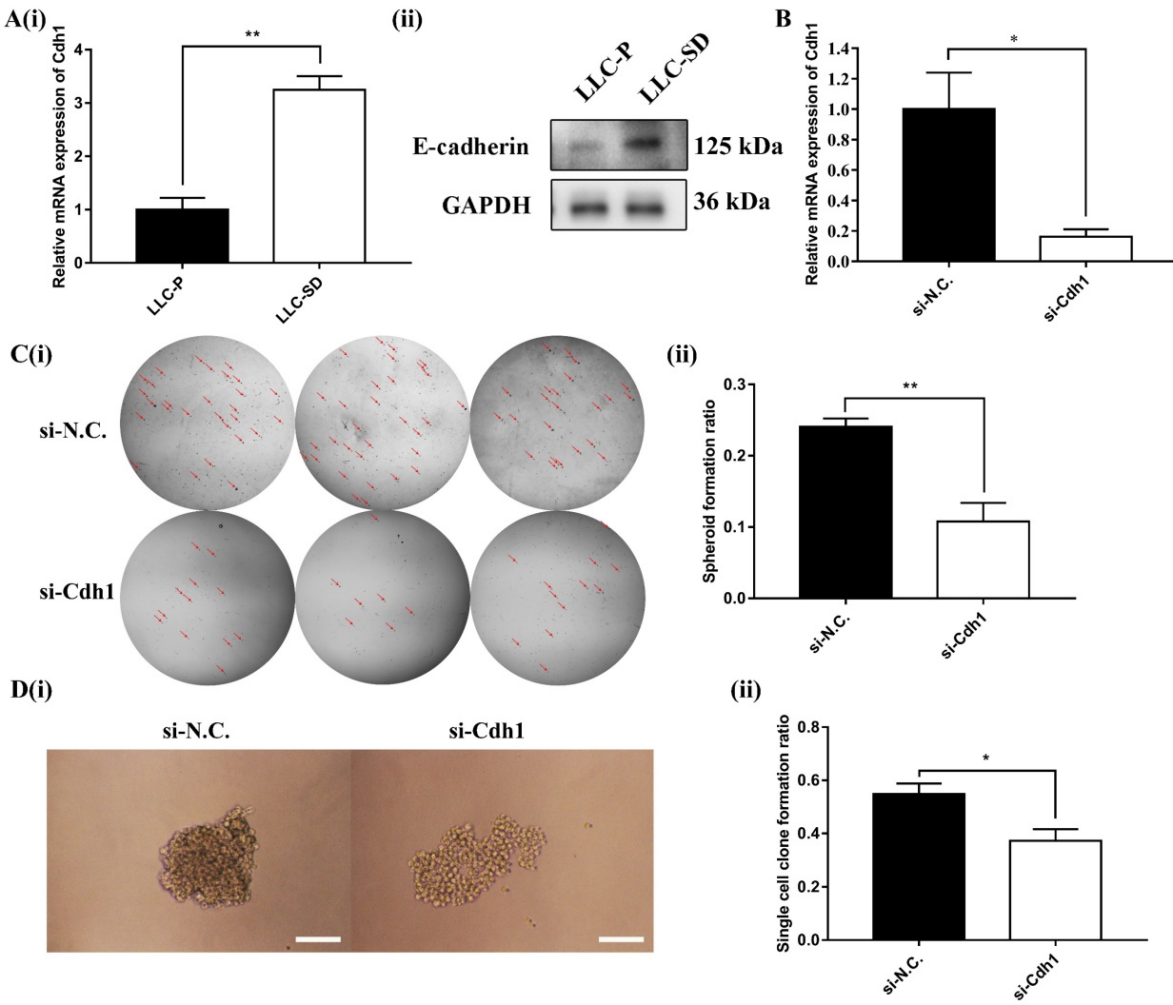
***Cdh1* regulates LLC-SD self-renewal *in vitro*. (A)(i)** mRNA expression of *Cdh1* in LLC-Parental and LLC-SD cells.** (ii)** Protein expression of *Cdh1* in LLC-Parental and LLC-SD cells. **(B)** mRNA expression of *Cdh1* in LLC-SD when the *Cdh1* was silenced, N.C. was the negative control si-RNA. **C(i)** The morphology of soft agar spheroid formation assay with crystal violet-stained using LLC-SD-siN.C. and LLC-SD-siCdh1 cells. Where the red arrow is pointing. **(ii)** Quantification of spheroid formation ratio. Data are presented as the mean ± S.E.M. of three independent experiments. **D(i)** The morphology of single-cell cloning formation from LLC-SD-siN.C. and LLC-SD-siCdh1 cells. Scale bars, 120μm.** (ii)** Quantification of single-cell cloning ratio. Data are presented as the mean ± S.E.M. of three independent experiments. (* *p* < 0.05, ** *p* < 0.01).

*Cdh1* encodes E-cadherin, which was shown to enhance the maintenance of embryonic stem (ES) cells pluripotency and self-renewal. However, it was unclear whether *Cdh1* may regulate the self-renewal of CSCs as it does in normal stem cells. Here, the expression of *Cdh1* in LLC-Parental and its CSC derivative LLC-SD cell line was detected by RT-qPCR and WB. And the results showed that LLC-SD CSC cells had elevated *Cdh1* mRNA (**Figure 2A-i**) and protein expression (**Figure 2A-ii**) in comparison with the LLC-Parental cells.

To investigate the relationship between elevated *Cdh1* and changes in the stem cell properties of LLC-SD cells, we inhibited *Cdh1* expression by transient siRNA interference and confirmed its effective silencing at the transcriptional level (**Figure 2B**). The effect of *Cdh1* silencing on stem cell self-renewal was measured by the soft agar colony formation assay and the single-cell cloning formation assay *in vitro*. Transient silencing of *Cdh1* expression in LLC-SD-siCdh1 cells significantly hindered the self-renewal capability (**Figure 2C**) and clonogenic efficiency (**Figure 2D**) of LLC-SD cells. These observations indicated that *Cdh1* promotes the self-renewal of LLC-SD lung CSC cells *in vitro*. Based on the bioinformatic analyses on the clinical datasets and these observations, we hypothesized that *Cdh1* may function as an oncogene by promoting the CSC stemness.

### Assessment of the function of *Cdh1* as an oncogene *in vivo*

In order to explore the role of oncogene of *Cdh1 in vivo*, we generated LLC-SD cell line in which *Cdh1* was stably and effectively inhibited using the lentivirus expression system (Methods, **Figure 5A and Figure 5B**). 10^3^ LLC-SD-shCdh1 and control cells were injected subcutaneously into each side of the hinder leg of nude mice, respectively. Measurable tumors were formed after 16 days of tumor cell inoculation (8/8 sites). Stable inhibition of *Cdh1* expression *in vivo* resulted in lower tumor incidence and smaller tumor burden compared with the control group (**Figure 5C and Figure 5D**).

Prior to this study, we have developed a clinically relevant syngeneic orthotopic mouse model of lung cancer which allowed the characterization of tumorigenicity and metastasis of LLC-SD CSC cells *in vivo* in C57BL/6 mice (Methods)^31^. Upon *Cdh1* stable depletion, 10^5^ LLC-SD-shN.C. cells and LLC-SD-shCdh1 cells were injected in the left lung of C57BL/6 mice, respectively. All mice were sacrificed on day 14 post tumor cell injection. Orthotopic tumor growth and metastatic progression in the thoracic cavity were examined and recorded. At the site of tumor cell injection, tumor nodules were developed in 8/8 mice injected with LLC-SD-shN.C. cells and only in 3/8 mice injected with LLC-SD-shCdh1 cells. In mice injected with LLC-SD-shN.C. cells, visible metastatic foci were found at right lung (1/8 mice), left thoracic cavity (5/8 mice), right thoracic cavity (1/8 mice) and mediastinal lymph nodes (3/8 mice). In contrast, no thoracic metastases were observed mice injected with LLC-SD-shCdh1 cells (**Table 2** and **Figure S1**). LLC-SD-shN.C. and LLC-SD-shCdh1 tumors on the lung were removed and analyzed by hematoxylin and eosin (HE) staining. Histology of the left lung (the orthotopic tumor) and the right lung (metastatic foci) from LLC-SD-shN.C. tumor showed destroyed alveoli and poorly differentiated hyperchromatic tumor cells with prominent nucleoli, as well as the presence of more severe right lung metastases. However, no typical lung carcinoma-like morphology was observed in the left lung tissue and no tumor cells were found in the right lung from LLC-SD-shCdh1 group (**Figure 5E-i** and** Figure 5E-ii**). Survival assay was also carried out and the time of death was recorded when it occurred until termination of the assay on Day 60 post tumor cell inoculation. Mice injected with LLC-SD-shN.C. cells died from 11th day until 27th day. Inhibition of *Cdh1* delayed the onset of death and prolonged the course of the survival assay which is evident from the right-shift of the survival curve (**Figure 5F**). Based on the tumorigenesis xenograft assay in nude mice, the orthotopic tumorigenesis and progression assay as well as the survival assay in the syngeneic mice, we conclude that *Cdh1* enhanced oncogenic properties of LLC-SD cells *in vivo*.

### Integrative bioinformatics analysis of *Cdh1*-associated signaling pathways in human lung adenocarcinoma

**Figure 3:**
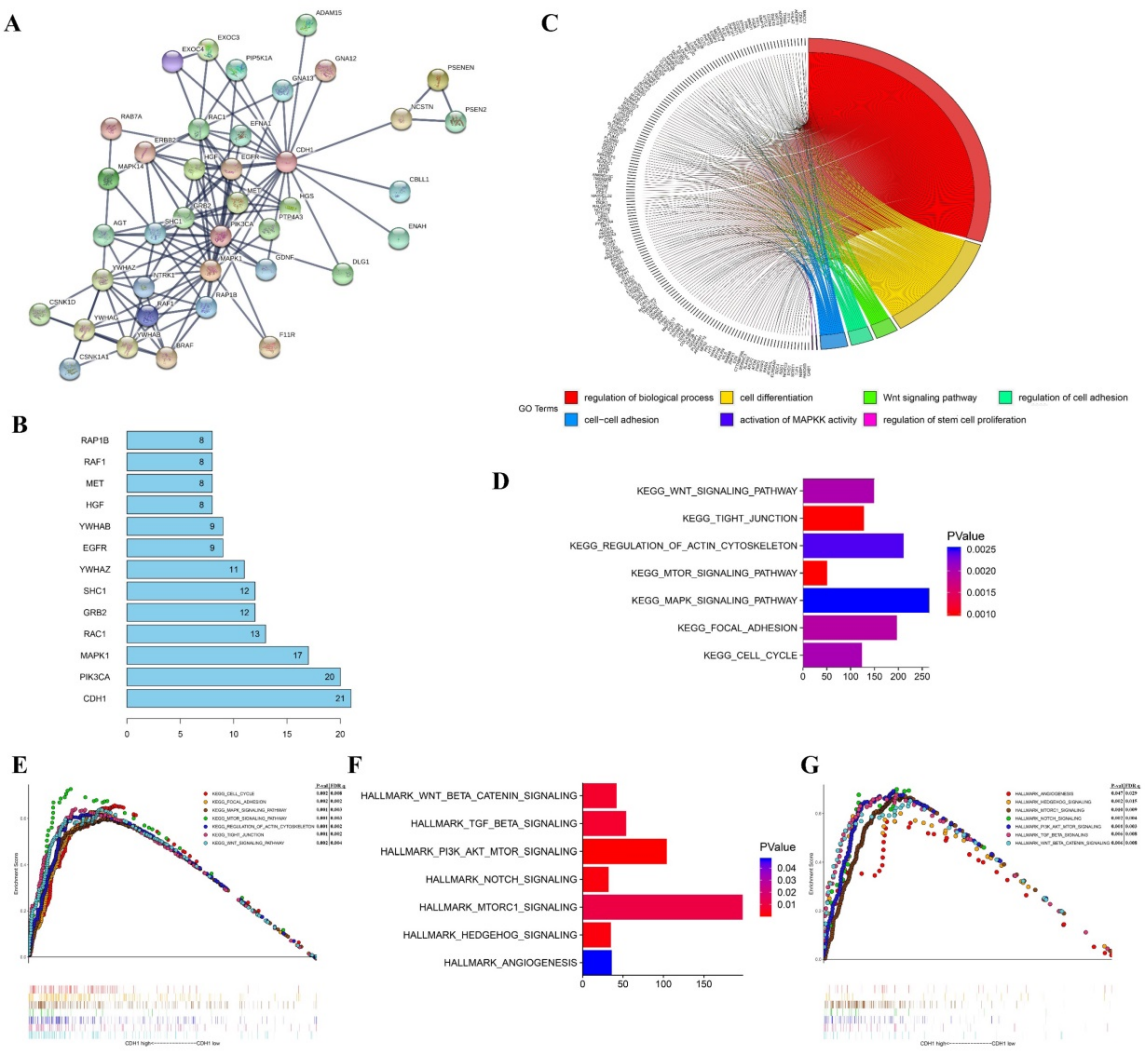
** The functions of* Cdh1* and genes significant associated with *Cdh1* were predicted by analysis of integrative bioinformatics in human lung adenocarcinoma. (A)** PPI network of the 50 most frequently altered neighbor genes associated with *Cdh1* from cBioportal. **(B)** Top 10 of key proteins from the PPI network. The vertical coordinate is the name of genes, and the horizontal coordinate represents the number of gene connections.** (C)** Chord plot displayed gene ontology (GO) terms of *Cdh1* co-expression genes in LUAD by KOBAS 3.0.** (D-E)** The GSEA results showed the KEGG pathway enrichment results using TCGA-LUAD datasets. **(F-G)** The GSEA results demonstrated the enriched hallmark gene sets results with correlation of *Cdh1* mRNA levels using TCGA-LUAD datasets.

To investigate the molecular mechanisms of the oncogene activity of *Cdh1* in human lung adenocarcinoma, we conducted additional bioinformatics analyses. We firstly acquired *Cdh1*-associated 50 most frequently altered neighbor genes from cBioportal databases. Then PPI analyses of these neighbor genes were performed in STRING (http://www.string-db.org/) to validate the protein interaction association to predict pathways likely mediating the oncogenic function of *Cdh1* carries in lung adenocarcinoma (**Figure 3A**). The results of the top 10 overall score of key proteins from PPI network demonstrated that *PIK3CA*, *MAPK1*, *EGFR* and* RAF1* represented key genes in these pathway (**Figure 3B**). Notably, these genes are key components of the oncogenic receptor tyrosine kinase-MAPK and PI-3K signaling pathways.

To explore the gene-enrichment and functional annotation analyses of *Cdh1*-related genes, we performed analysis of Gene Ontology (GO) and Gene Set Enrichment Analysis (GSEA) (Methods). The results from GO analysis revealed that target genes in the LUAD group mainly participated in regulation of biological process (GO:0050789), cell differentiation (GO:0030154), Wnt signaling pathway (GO:0016055), regulation of cell adhesion (GO:0030155), cell-cell adhesion (GO:0098609), activation of MAPKK activity (GO:0000186) and regulation of stem cell proliferation (GO:0072091) (**Figure 3C** and** Table 1**). The results of Kyoto Encyclopedia of Genes and Genomes (KEGG) analysis indicated that *Cdh1*-related genes were enriched in the following pathway in LUAD group, including Wnt signaling pathway, Tight junction, regulation of actin cytoskeleton, mTOR signaling pathway, MAPK signaling pathway, focal adhesion and cell cycle pathway (**Figure 3D** and** Figure 3E**) which are well characterized oncogenic pathways for human cancer, including lung cancer. We then evaluated the oncogenic pathway more likely to be *Cdh1*-related by GSEA, listing those genes in the categories described as hallmarks of gene expression and found that they are related to Wnt signaling pathway, TGF-β signaling pathway, PI3K signaling pathway, Notch signaling pathway and Hedgehog signaling pathway (**Figure 3F** and** Figure 3G**).

Taken together, bioinformatics analysis results collectively predicted the close association of the oncogenic pathways, such as the PI3K and MAPK pathways and the oncogene phenotypes of *Cdh1* in human LUAD. Based on the findings of these analyses and the promotion of self-renewal in LLC-SD CSCs, we next explored the possibility of a cross-talk between the oncogenic pathways and the stem cell pathway as the mechanism that underlies the oncogenic function of *Cdh1*.

### The PI3K and MAPK signaling pathways had an opposing effect on *Cdh1* expression and its regulation of CSC self-renewal *in vitro*

**Figure 4:**
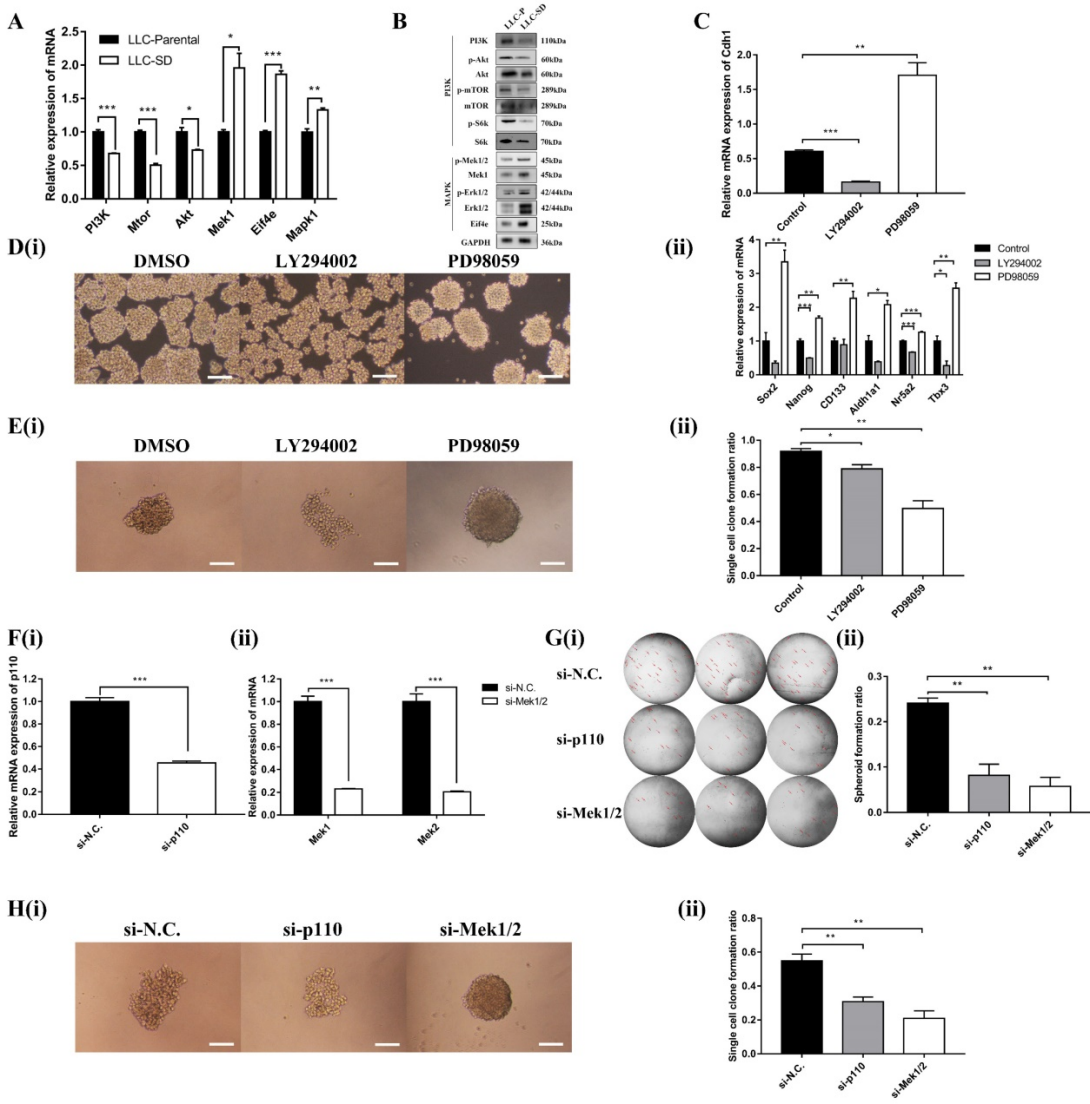
** The PI3K and MAPK signaling pathway mediate *Cdh1* regulation of CSC self-renewal *in vitro*. (A)** mRNA expression and **(B)** Protein expression of related genes of PI3K and MAPK signaling pathways in LLC-Parental and LLC-SD cells. **(C)** mRNA expression of *Cdh1* in LLC-SD cells with treatment of LY294002 and PD98059 by RT-qPCR. DMSO was used as control. **D(i)** The morphology of spheroid formation in LLC-SD cells treated with LY294002 and PD98059. DMSO was used as control. Scale bars, 120μm. **(ii)** Expression of stem genes in LLC-SD cells by RT-qPCR, the DMSO was used as control. **E(i)** The morphology of single-cell cloning formation in LLC-SD cells treated with LY294002 and PD98059. DMSO was used as control. Scale bars, 120μm. **(ii)** Quantitative analysis of single-cell cloning ratio. Data are presented as mean ± S.E.M. of three independent experiments. **(F)(i-ii)** Efficiency of p110 and Mek1/2 siRNAs (si-p110 and si-Mek1/2) interference determined by RT-qPCR. **G(i)** The morphology of soft agar spheroid formation assay with crystal violet-stained using LLC-SD-siN.C., LLC-SD-sip110 and LLC-SD-siMek1/2 cells. Where the red arrow is pointing.** (ii)** Quantification of spheroid formation ratio. Data are presented as mean ± S.E.M. of three independent experiments. **H(i)** The morphology of single-cell cloning formation in LLC-SD upon p110 and Mek1/2 knock down, LLC-SD-siN.C. was the negative control. Scale bars, 120μm. **(ii)** Quantification of single-cell cloning efficiency in which 96 wells was analyzed. Data are presented as mean ± S.E.M. of three independent experiments. (* *p* < 0.05, ** *p* < 0.01, *** *p* < 0.001).

We first analyzed mRNA and protein expression of key signaling components of the PI3K and MAPK pathway by RT-qPCR and WB in LLC-Parental and LLC-SD cell lines which differs in *Cdh1* expression. The results showed that the expression of the signaling molecules on the MAPK signaling pathway including *Mapk1*, *Mek1* and *Eif4e* was robustly upregulated in LLC-SD cells, while the expression of signaling molecules of the PI3K pathway including *PI3K*, *Akt* and* Mtor* was higher in LLC-Parental cells (**Figure 4A**). Altered protein expression by confirmed by WB was in agreement with the RT-qPCR results (**Figure 4B**). This set of observations indicate that in LLC-SD CSC cells, while the MAPK pathway is upregulated in the LLC-SD cells, the PI3K pathway is inhibited.

We next examined whether MAPK and/or PI3K activation are required for the *Cdh1* expression, LLC-SD cells were treated with PI3K inhibitor LY294002 or MEK inhibitor PD98059, respectively. Shown in **Figure 4C**, inactivation of PI3Kand MAPK pathway by their respective synthetic inhibitor had an opposite effect on *Cdh1* expression. LY294002 markedly reduced *Cdh1* expression, whereas PD98059 significantly increased *Cdh1* levels. Meanwhile, LLC-SD cells exhibited the irregular morphology with a loose structure after the inhibition of PI3K signaling pathway by LY294002 (**Figure 4D-i**). Conversely, LLC-SD cells exhibited the stem-like spheroid morphology with a compact structure after the inhibition of MAPK signaling pathway by PD98059 (**Figure 4D-i**). We further examined the expression of the candidate stemness genes, *Sox2*, *Nanog*, *CD133*, *Aldh1a1*, *Nr5a2* and *Tbx3* by RT-qPCR. And the results demonstrated that the expression of *Nanog*, *Nr5a2* and *Tbx3* were significantly lower in the LLC-SD treated with LY294002, whereas the expression of *Sox2*, *Nanog*, *CD133*, *Aldh1a1*, *Nr5a2* and *Tbx3* were highly upregulated in the LLC-SD treated with PD98059 than that in the negative control (**Figure 4D-ii**). The single-cell cloning assay in 96-well plate showed that colonies treated with LY294002 exhibited loosely irregular morphology and the colonies treated with PD98059 exhibited regular compact spheroid (**Figure 4E-i**). However, the single-cell cloning rate of SD cells with inhibitors treatment were both lower than that of control cells. This may have to do with inhibition of both self-renewal and proliferation activities upon inhibition of the PI3K and MAPK signaling (**Figure 4E-ii**).

Subsequently, successful PI3K silencing by targeting the* p110* catalytic unit and *Mek1/2* silencing by siRNA was confirmed by RT-qPCR analysis (**Figure 4F-i** and **Figure 4F-ii**). The effect of *p110* and *Mek1/2* silencing on the stemness was measured by the soft agar colony formation assay and the single cell cloning assay and yielded very comparable findings (**Figures 4G and H**) as using the inhibitors (**Figure 4E**). Taken together, our data supports that inactivation of the MAPK pathway and activation of the PI3K pathway correlates with *Cdh1* up-regulation in LLC-SD cells and *Cdh1* promotes self-renewal in LLC-SD cells.

### The PI3K and MAPK signaling pathway mediates oncogenesis of LLC-SD cells* in vivo*

**Figure 5:**
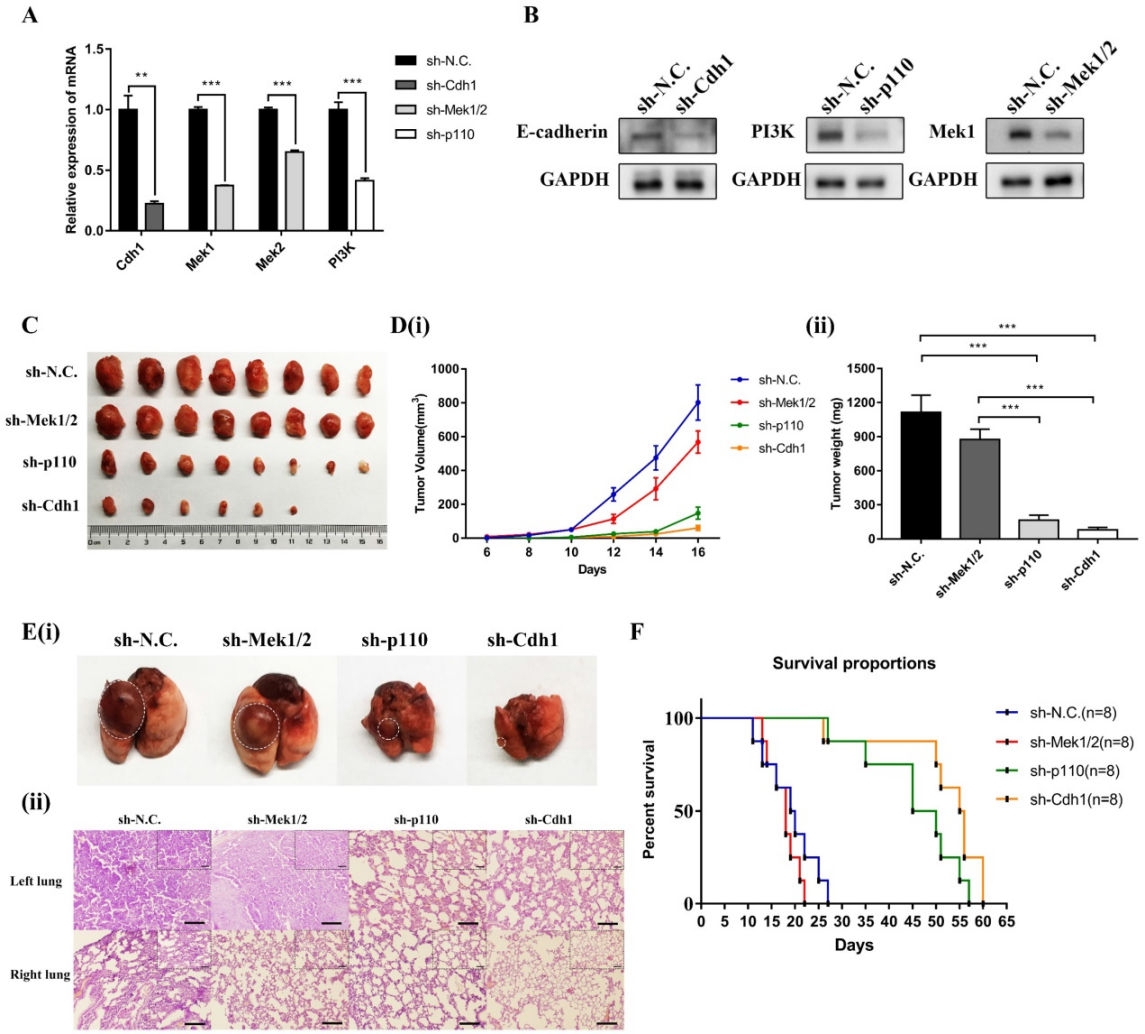
** The PI3K and MAPK signaling pathways mediate LLC-SD oncogenesis* in vivo.* (A)** The analysis of mRNA expression of shRNA in the three experimental groups of LLC-SD cells by RT-qPCR, sh-N.C. was the negative control, sh-Cdh1 was the stable silencing of *Cdh1*, sh-Mek1/2 was the silencing of *Mek1* and *Mek2*, and sh-p110 was the silencing of *p110*. **(B)** Efficiency of shRNAs (sh-Cdh1, sh-Mek1/2 and sh-p110) interference determined by Western blot analysis. **(C)** Tumor formation in nude mice following injection of control cells and genetically modified LLC-SD (sh-Cdh1, sh-Mek1/2 and sh-p110) cells respectively. **(D)** Tumor growth curves of LLC-SD-shRNAs (sh-Cdh1, sh-Mek1/2 and sh-p110) and control cells (sh-N.C.) in nude mice.** (i)** Tumor volume. **(ii)** Tumor weight. **E(i)** Images of left lung orthotopic nodules in C57BL/6 mice injected with LLC-SD-shRNAs (sh-Cdh1, sh-Mek1/2 and sh-p110) or control cells.** (ii)** Immunohistochemistry analysis of the left and right lung harvested from C57BL/6 mice injected with control or experimentally modified LLC-SD cells (sh-Cdh1, sh-Mek1/2 and sh-p110) respectively. Scale bars = 120μm, the black box indicates the enlarged area, bar = 60μm. **(F)** The survival curve of C57BL/6 mice injected with 1×10^5^ control and LLC-SD-shRNAs (sh-Cdh1, sh-Mek1/2 and sh-p110) cells respectively. (** *p* < 0.01, *** *p* < 0.001).

Given the essential role of PI3K and MAPK signaling pathway mediated *Cdh1* in regulating LLC-SD self-renewal *in vitro*, we first generated LLC-SD derivative cell lines in which *p110* and *Mek1/2* expression was stably inhibited by RNA interference (sh-p110 and sh-Mek1/2). Effective inhibition of *p110* and *Mek1/2* expression was confirmed by RT-qPCR (**Figure 5A**) and western blot (**Figure 5B**). Then we conducted xenograft transplantation assay in nude mice to investigate the importance of MAPK and PI3K pathway in LLC-SD lung cancer tumorigenesis. 10^3^ LLC-SD-shMek1/2, LLC-SD-shp110 and LLC-SD-shN.C. cells were injected subcutaneously into each side of the hinder leg of nude mice respectively. The lower tumor incidence and smaller tumor burden were measured by tumor growth and tumor weight *in vivo* upon inactivation of the PI3K pathway by *p110* knockdown. However, inhibition of* Mek1/2* expression and the resultant inactivation of the MAPK pathway produced no significant difference in terms of tumor volume and weight compare with LLC-SD-shN.C. cells (**Figure 5C and Figure 5D**).

As for orthotopic tumor transplantation of C57BL/6 mice assay, 10^5^ LLC-SD-shN.C. cells, LLC- SD-shMek1/2 and LLC-SD-shp110 cells were injected in the left lung of C57BL/6 mice, respectively. All mice were sacrificed on day 14 post tumor cell injection. The orthotopic tumor growth and metastatic progression in the mediastinal lymph nodes and thoracic cavity showed no significant difference in LLC-SD-shMek1/2 cells than that of LLC-SD-shN.C. cells. However, it was reduced upon inactivation of the PI3K pathway by *p110* knockdown, which was consistent with the LLC-SD-shCdh1 group cells (**Table 2** and **Figure S1**). Histology of the left lung (the orthotopic tumor) and the right lung (metastatic foci) confirmed the presence of more severe right lung metastases in mouse received LLC-SD-shN.C. and LLC-SD-shMek1/2 cells (**Figure 5E**). Survival assay was carried out and the time of death was recorded when it occurred. While the survival of mice receiving *Mek1/2* knockdown LLC-SD cells and LLC-SD-shN.C. cells were comparable and mice incurred rapid death. In contrast, inactivation of PI3K by *p110* silencing delayed the onset of death and prolonged the course of the survival as evident by the right-shifted survival curve. Inactivation of PI3K produced similar effect on *in vivo* survival as seeing in *Cdh1* silenced LLC-SD- shCdh1 group (**Figure 5F**). These sets of observations are consistent with the *in vitro* findings, confirming the importance of PI3K and MAPK pathway of CSC self-renewal regulation in oncogenesis and tumor progression *in vivo*.

## Discussion

In cancer literature, the role of E-cadherin, encoded by* Cdh1* gene, that functions as a tumor suppressor is widely reported. Downregulation or loss of the E-cadherin is reported to be involved in the invasion and metastatic progression of many malignancies 20, 35-37. The main mechanism underlying the tumor suppression function of *Cdh1* is the inhibition of epithelial-mesenchymal transition (EMT), a key process for tumor progression 17. However, small bodies of reported studies have demonstrated the pro-oncogenic activity of *Cdh1*.* Cdh1* upregulation and the subsequent promotion of the mesenchyme-to-epithelial transition (MET) for colonization phase of metastasis have been implicated 23-25. In addition, our knowledge of the promoting effect of *Cdh1* on stem cell self-renewal comes mostly from studies involving normal stem cells 14-16. A recent study reported that *Cdh1* can substitute for *Oct4* during somatic cell reprogramming and is required to maintain the undifferentiated state of mouse embryonic stem cells (mESCs) 12. However, the role of *Cdh1* in regulating CSCs function has not been established prior to the present study 26, 38. This study has made the following novel findings that an alternative mechanism underlying the oncogenic activity of *Cdh1* may be realized via its promotion of CSC self-renewal, similar to its function in normal stem cells:

First, the oncogene function of *Cdh1* overexpression in most cancers including lung adenocarcinoma has been uncovered by bioinformatic analysis and data mining of publically available cancer clinical databases, including GEPIA, Oncomine, TCGA, GEO and Kaplan-Meier plotter. Prior to this study, the clinical relevance of *Cdh1* has not been well explored and established. *Cdh1* expression is elevated in 17/33 human cancer types anaylazed, including lung adenocarcinoma (**Figure 1A-F**). Thus, high *Cdh1* expression is a rather general feature of human cancer and is not limited to lung cancer. In depth analysis of lung adenocarcinoma data sets show the diagnostic and prognostic significance of *Cdh1* high expression which merits further investigation to establish whether it is a new prognostic marker for lung cancer. We also found that a low *Cdh1* expression may be a protective factor in patients with LUAD (**Figure 1G**). Further in-depth mechanistic characterization will answer whether *Cdh1* is a novel target for therapeutic development.

Second, utilizing the stable lung CSCs cellular models and syngeneic orthotopic lung cancer model we established and characterized^31^, ^39^, ^40^, we provide convincing experimental evidence supporting a rather definitive role of *Cdh1* in regulating self-renewal of LLC-SD *in vitro* (**Figure 2**) as well as promoting lung cancer oncogenesis and progression* in vivo* (**Figure 5, Figure S1 and Table 2**). These observations provide new mechanistic understanding other than EMT/MET to confer the oncogenic activity of *Cdh1* by regulating the self-renewal of CSCs.

Third, this study was the first attempt to predict the potential molecular mechanisms underlying the oncogenic activity of *Cdh1* in LUAD using bioinformatics analyses. We performed PPI analyses on *Cdh1*-associated 50 most frequently altered neighbor genes from cBioportal databases and demonstrated that* PIK3CA*, *MAPK1*, *EGFR* and* RAF1* served as core genes in the *Cdh1* PPI network (**Figure 3A-B**). Furthermore, GO, KEGG and Hallmarks gene sets analysis showed that the *Cdh1*-associated 50 genes were mostly associated with PI3K and MAPK signaling pathways in caner (**Figure 3C-G** and** Table 1**). PI3K/AKT and MAPK signaling pathways are the two most important intracellular signal transduction pathways that are activated to mediate the activity of receptor tyrosine kinases 41-43. These two signaling pathways also regulate normal stem cell self-renewal and differentiation 44, 45. We confirmed these predictions with experimentation.

Fourth, we have identified and verified an intricate cross-talk between the oncogenic pathways and stem cell pathway using our stable lung CSCs model both* in vitro* and *in vivo*. We provided convincing evident for the positive regulation of PI3K and negatively regulation of MAPK on *Cdh1* in the maintenance of lung CSC self-renewal and stem cell properties (**Figure 4**). Further, using the nude mouse xenograft and orthotopic lung transplantation model, characterized the effect of this cross-talk on promoting lung cancer oncogenesis and progression *in vivo* (**Figure 5** and** Table 2**). Noteworthy, the inconsistency of function experiments upon MAPK inhibition *in vitro* and *in vivo* might be ascribed to in the growing LLC-SD tumors, inactivation of MAPK pathway lead to the concomitant inhibition of both stem cell self-renewal and the proliferative activity of the LLC-SD cells for tumor expansion. Moreover, there might be a mechanism for feedback regulation between *Cdh1* and PI3K and MAPK signaling 46, 47. This merits further investigations.

In conclusion, to the best of our knowledge, our study is the first to show that *Cdh1* functions as an oncogene by inducing self-renewal of lung cancer stem-like cells via the opposing effect of PI3K and MAPK signaling pathways. Therefore, these results show the potential of *Cdh1* as a new clinical target for diagnosis, prognosis and treatment of LUAD which will be explored in our future studies.

## Supplementary Material

Supplementary figures and tables.

## Figures and Tables

**Table 3:** Primers for RT-qPCR

Gene name	Forward primers	Reverse primers
mouse *Cdh1*	AGCCATTGCCAAGTACATCC	TCTGGCCTGTTGTCATTCTG
mouse *PI3K*	GCAGAGGGCTACCAGTACAGA	CTGAATCCAAGTGCCACTAAGG
mouse *Mtor*	AGACCTTGAGTTGGCTGTGC	CCTCTGCTTGGATGTGATGA
mouse *Akt*	ACTCATTCCAGACCCACGAC	CACAATCTCCGCACCATAGA
mouse *Mek1*	GTGCAGTCGGACATCTGGAG	CCACATGGCATCCAAACAGT
mouse *Mek2*	ACATGGATGGTGGCTCACTG	CTGGTGCTTCTCTCGGAGGT
mouse *Eif4e*	TGTGGGTAGCAGAGTGGAAA	CAACAAGAGCAGGCGGTTAT
mouse* Mapk1*	CTTCCAACCTCCTGCTGAAC	TCTGTCAAGAACCCTGTGTGA
mouse* Sox2*	AGGGCTGGGAGAAAGAAGAG	ATCTGGCGGAGAATAGTTGG
mouse *Nanog*	TTAGAAGCGTGGGTCTTGGT	CCCTCA AACTCCTGGTCCTT
mouse *CD133*	CTCCCATCAGTGGATAGAGAACT	ATACCCCCTTTTGACGAGGCT
mouse *Aldh1a1*	ATACTTGTCGGATTTAGGAGG CT	GGGCCTATCTTCCAAATGAAC A
mouse *Nr5a2*	AAACGGGCAGTAACCCTCTT	CCACATTTCAGCAACAGCAG
mouse *Tbx3*	CGGAAGTCCCATTATCCTCA	CCCTCTACAAGCGCTCAGAT
mouse *p110*	TAAAGGCCGAAAGGGTGCTA	GCGGTACAGGCCAGAGATTC
mouse *Tbp*	AGGGATTCAGGAAGACCACA	ATGCTGCCACCTGTAACTGA

**Table 1:** Enriched GO terms of the genes co-expressed with *Cdh1*

GO ID	Term	Count	P-value
GO:0050789	regulation of biological process	157	4.84E-40
GO:0030154	cell differentiation	60	1.73E-14
GO:0016055	Wnt signaling pathway	12	2.42E-05
GO:0030155	regulation of cell adhesion	12	3.61E-04
GO:0098609	cell-cell adhesion	14	6.48E-04
GO:0000186	activation of MAPKK activity	2	0.038452263
GO:0072091	regulation of stem cell proliferation	2	0.047918442

**Table 2:** Characterization of orthotopic LLC-SD tumorigenesis and metastatic progression

10^5^ cells(n=8)	*In situ* Metastasis				
	Lung(left)	Mediastinal lymph	Lung(right)	Thoracic cavity(left)	Thoracic cavity(right)
sh-N.C.	8/8	3/8	1/8	5/8	1/8
sh-Mek1/2	8/8	4/8	0/8	3/8	1/8
sh-p110	3/8	0/8	0/8	0/8	0/8
sh-Cdh1	3/8	0/8	0/8	0/8	0/8
