# Supplementary material for: Cdh1 functions as an oncogene by inducing self-renewal of lung cancer stem-like cells via oncogenic pathways
Source: Int J Biol Sci. 2020 Jan 1;16(3):447–59. doi: 10.7150/ijbs.38672 (PMC6990901; doi:10.7150/ijbs.38672)
Supplement: Supplementary file 1 — Supplementary figures and tables. [file ijbsv16p0447s1.pdf]

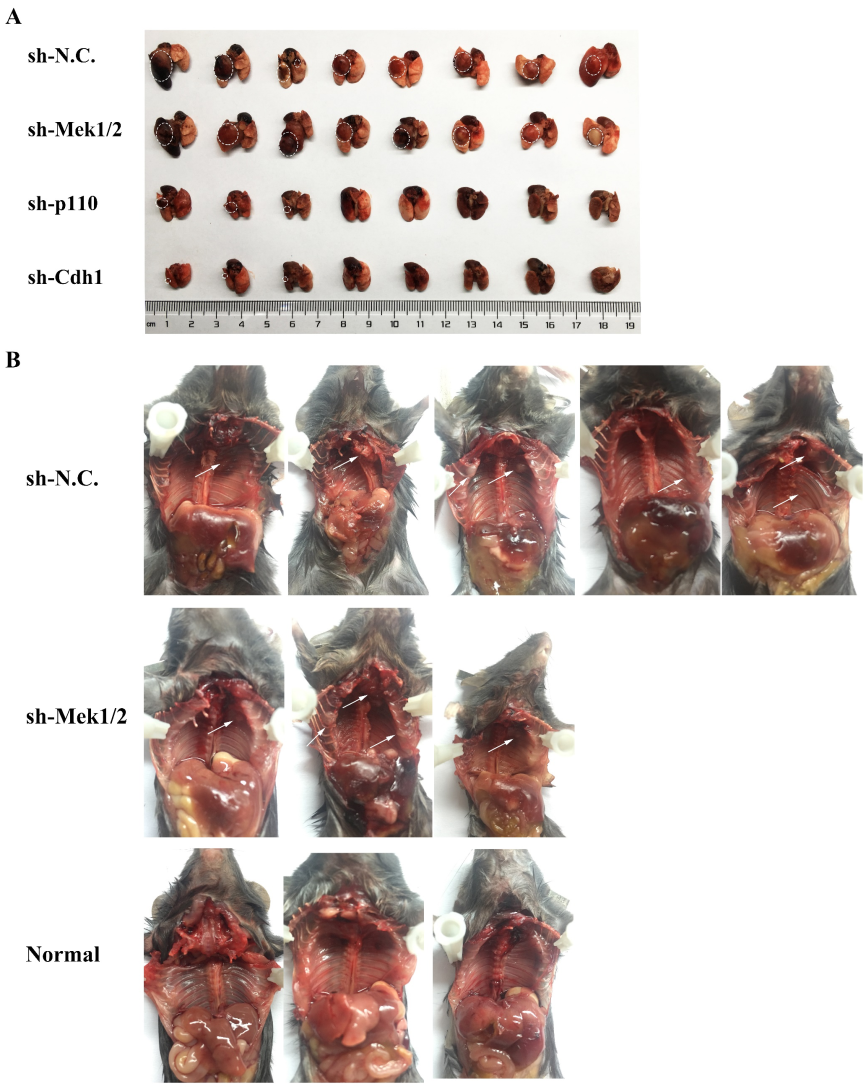

FIGURE S1 Orthotopic lung tumorigenesis and progression. (A) Images of orthotopic tumor transplantation in C57BL/6 mice. **(B)** Images of normal and thoracic cavity metastases upon orthotopic tumor transplantation in C57BL/6 mice.
